# Supplementary material for: The genomic architecture of circulating cytokine levels points to drug targets for immune-related diseases
Source: Commun Biol. 2025 Jan 10;8:34. doi: 10.1038/s42003-025-07453-w (PMC11724035; doi:10.1038/s42003-025-07453-w)
Supplement: Supplementary file 2 — Supplementary information [file 42003_2025_7453_MOESM2_ESM.pdf]

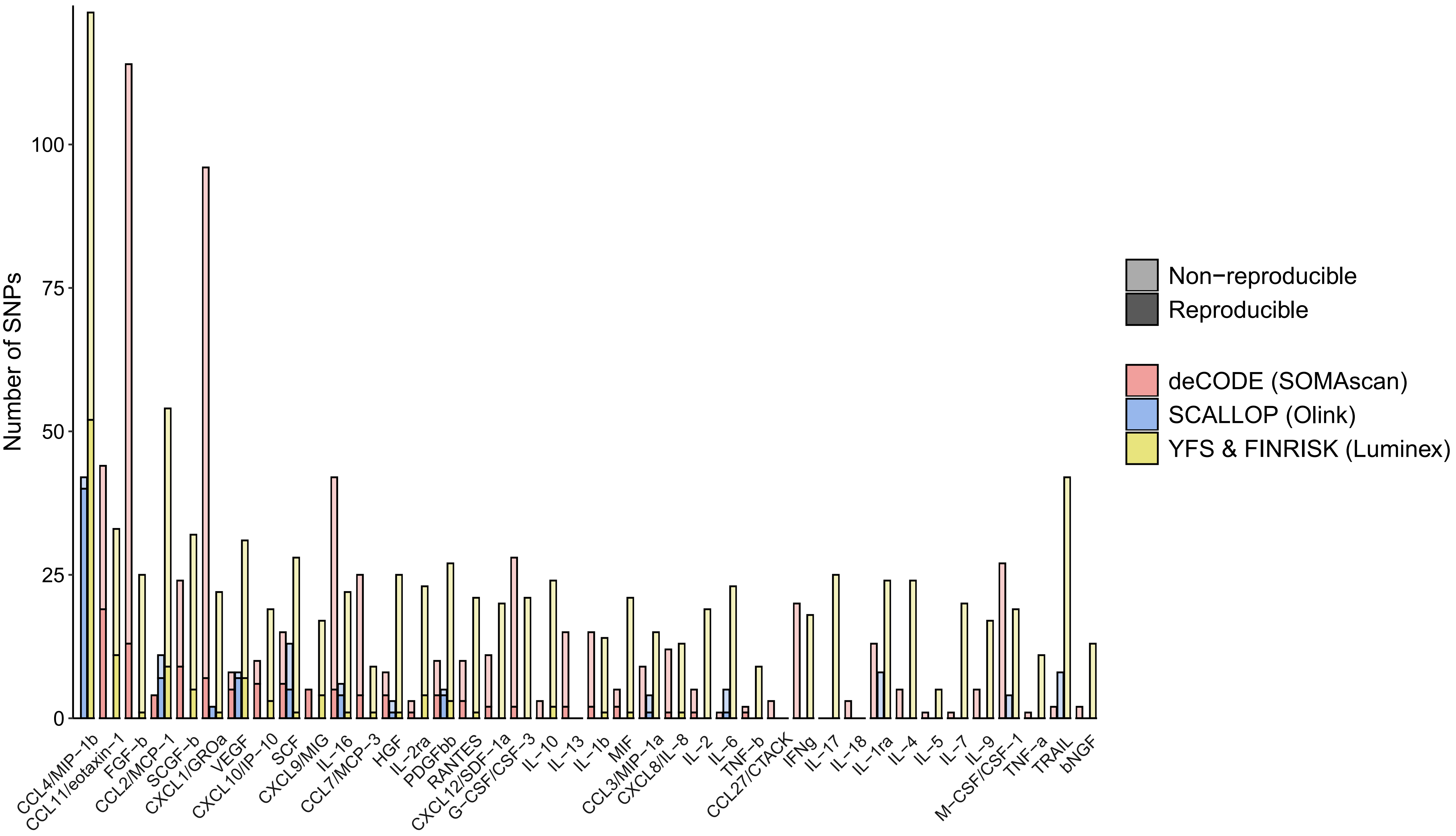

Supplementary Figure S1. Comparisons of significant genomic loci for 40 circulating cytokines across 3 proteomics assays. Number of reproducible and non-reproducible SNPs per cytokine (depicted as saturated and light-colored bars, respectively) for deCODE, SCALLOP and YFS & FINRISK cohorts ordered from high to low with respect to the total number (across databases) of replicated SNPs. The saturated part of the bars represents the number of SNPs replicated in both of the other cohorts, where replicated SNPs are defined as those confined to significant loci (p-value < 0.05) and directionally concordant. Colored bars represent deCODE consortium in red, SCALLOP consortium in blue and YFS & FINRISK cohorts in yellow. SNP, Single-nucleotide polymorphism; SCALLOP, Systematic and Combined AnaLysis of Olink Proteins; YFS & FINRISK, Cardiovascular Risk in Young Finns Study.

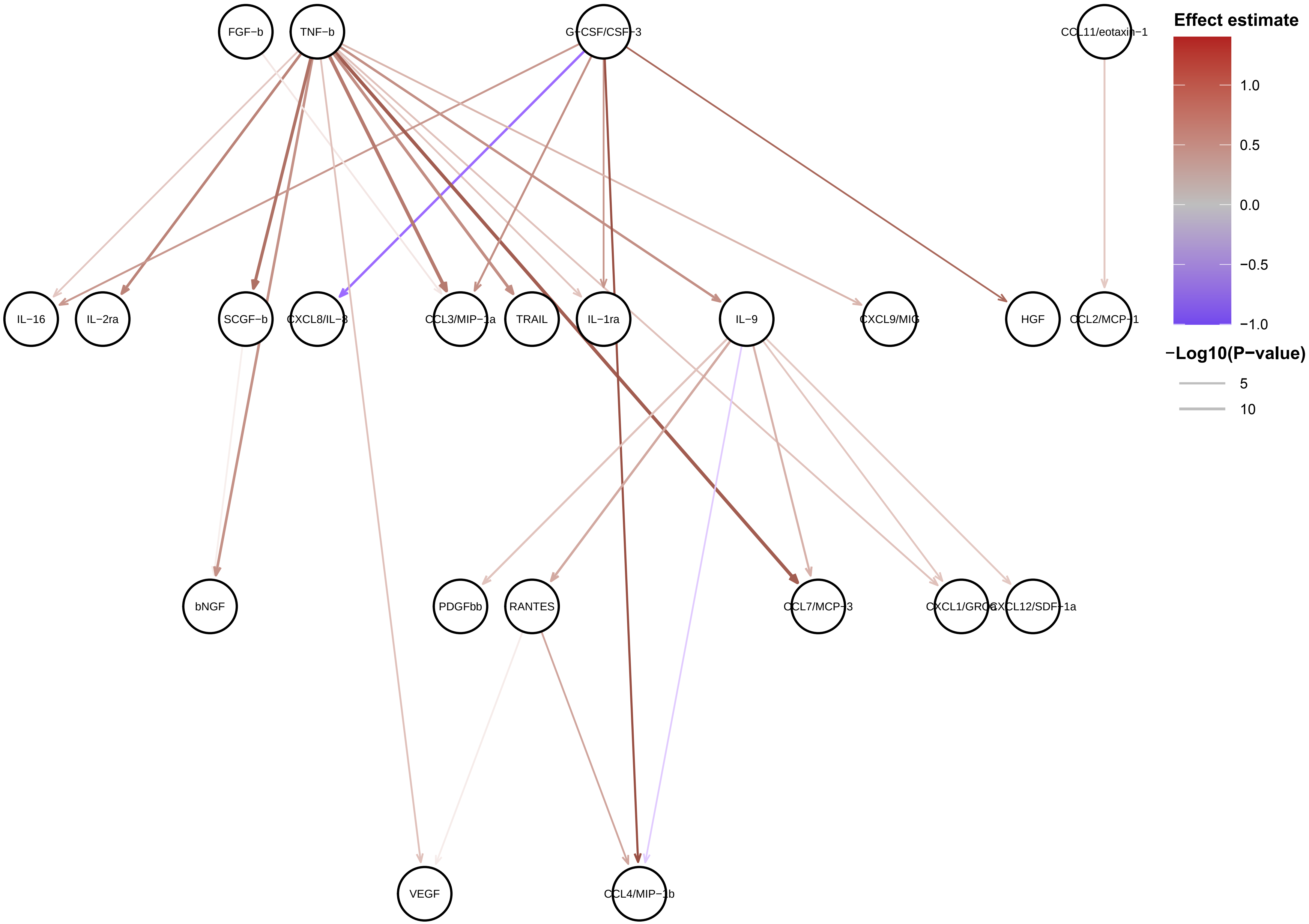

Supplementary Figure S2. Cross-cytokine genetic associations. Cis-Mendelian randomization excluding heterogenic variants (Het P-value < 0.05) and variants associated with the exposure and outcome in the instruments lists between genetically proxied circulating cytokine levels. Arrow heads show the direction of causal influence, color gradient indicates the effect estimate and line width the logarithm-adjusted Benjamin-Hochberg corrected significance level.

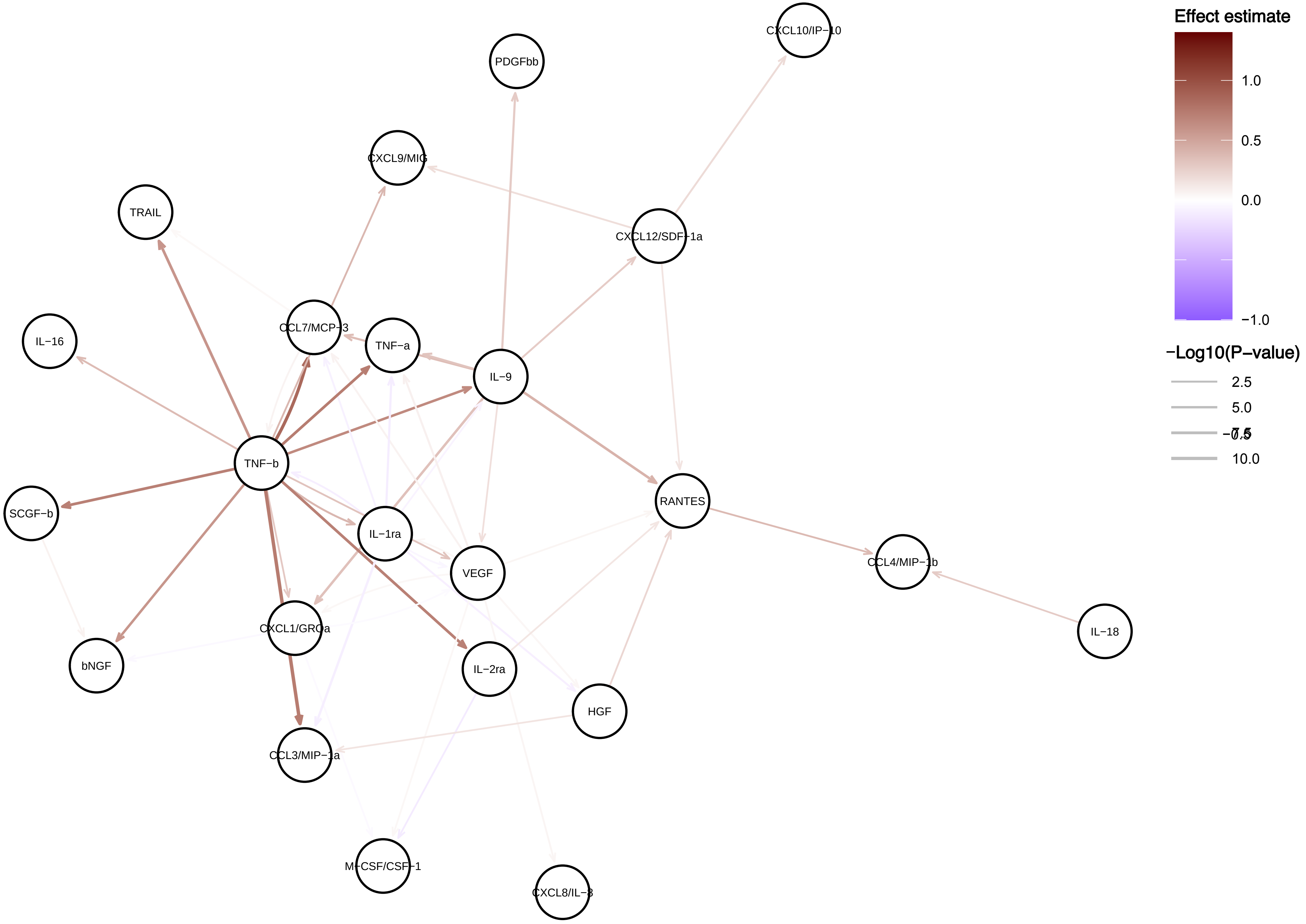

Supplementary Figure S3. Cross-cytokine genetic associations. Cis-Mendelian randomization excluding variants that were more strongly associated with the "outcome" than the "exposure" cytokine (Steiger filtering) in the instruments lists between genetically proxied circulating cytokine levels. Arrow heads show the direction of causal influence, color gradient indicates the effect estimate and line width the logarithm-adjusted Benjamin-Hochberg corrected significance level.

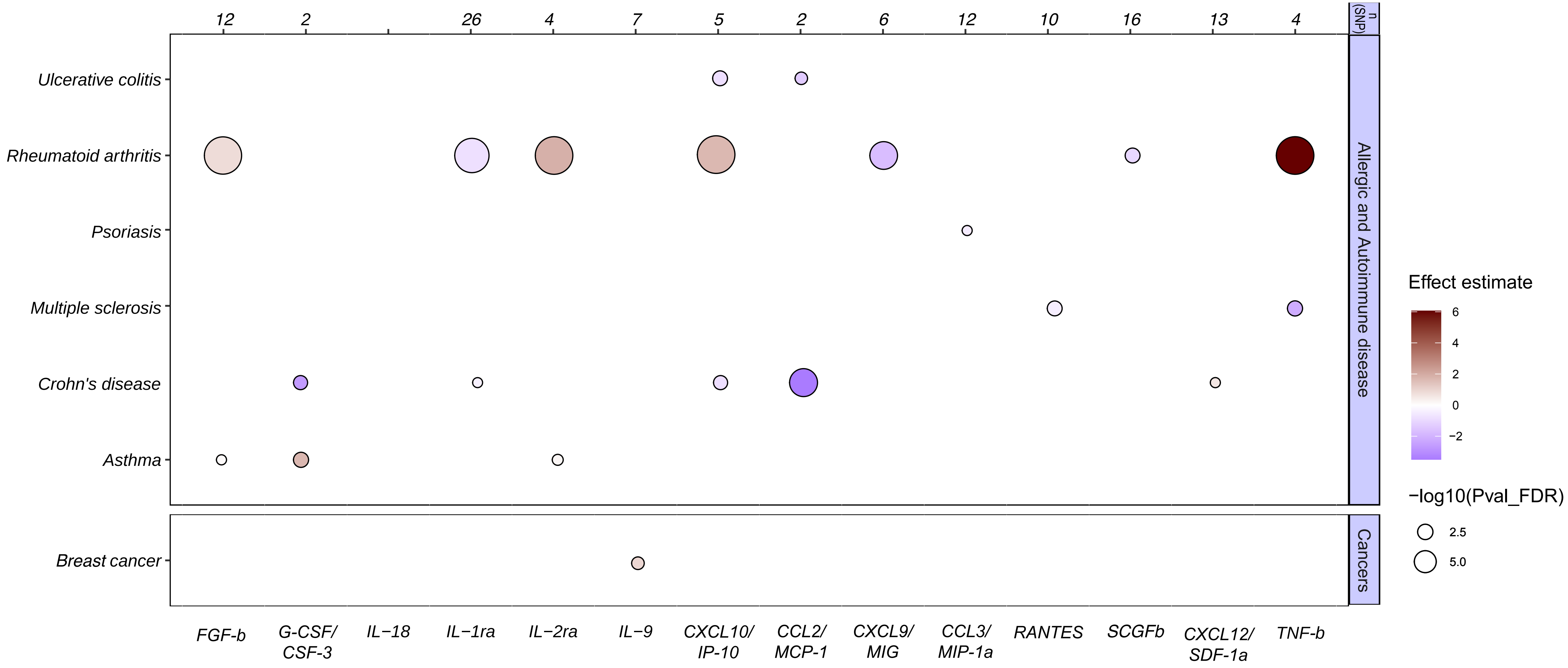

Supplementary Figure S4. Cis-Mendelian randomization analyses between genetically proxied cytokine levels and disease risk. Significant associations between circulating cytokine levels excluding heterogenic variants (Het P-value < 0.05) in the instruments lists are shown for allergic and autoimmune, and cancer outcomes. Effect sizes and log-transformed, Benjamin-Hochberg corrected p-values are illustrated by color gradient and circle size, respectively. Only cytokines and disease endpoints with at least 1 significant association are depicted. n(SNP), indicates average number (across disease outcomes) of cis-acting genetic variants used as instruments in MR analyses.

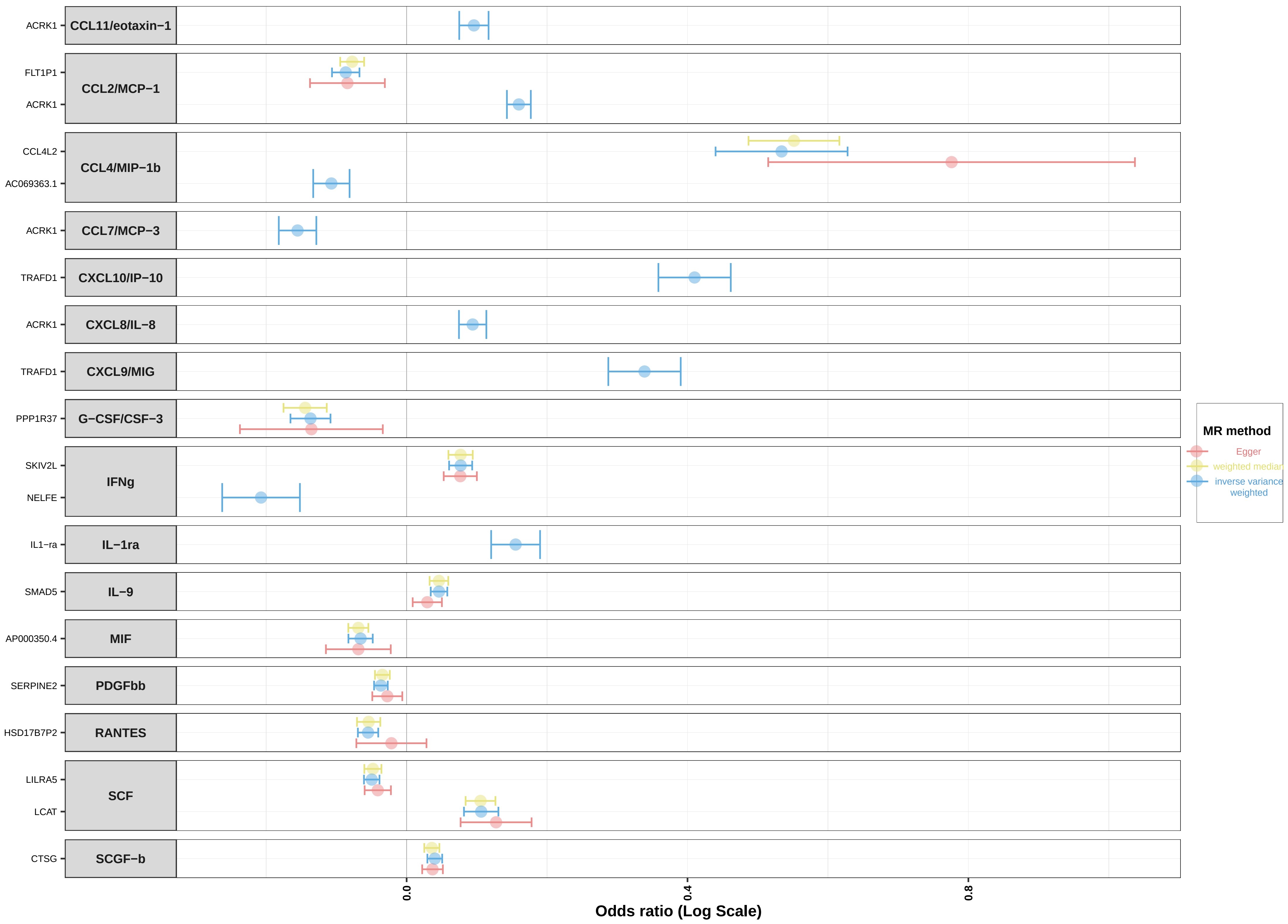

Supplementary Figure S5. Mendelian randomization-based transcriptome wide association study between expressive trait loci and circulating cytokine levels. TWAS-MR results including Egger, weighted median, inverse variance weighted MR methods are depicted for the top line results (log10 P-value > 10). MR methods are color-coded. Sensitivity analyses showed directional concordance with the main MR approach for 77% and 76% of the associations calculated with weighted median MR and MR Egger, respectively. Missing MR parameters were due to an insufficient number of valid instruments (weighted median MR) or weak associations between instruments and exposure (MR Egger). TWAS-MR, Mendelian randomization-based transcriptome wide association study. Error bars and circles represent mean and 95% confidence intervals, respectively.

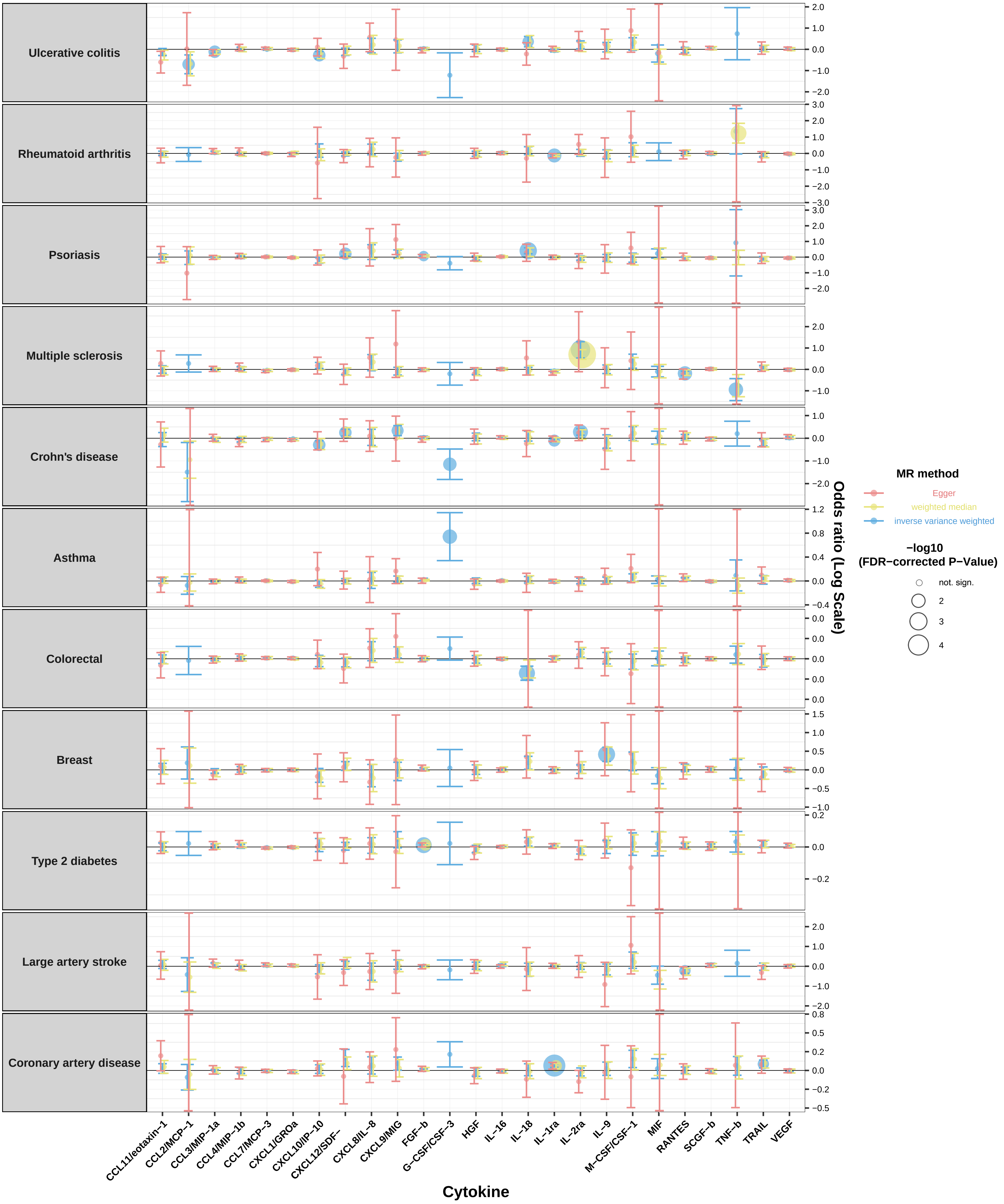

Supplementary Figure S6. Drug-target mendelian randomization analyses between genetically proxied cytokine levels and disease risk. Associations between circulating cytokine levels and allergic and autoimmune, cardiometabolic and cancer outcomes are shown for Egger, weighted median, inverse variance weighted MR methods. MR methods are color-coded, log-transformed, Benjamin-Hochberg corrected p-values are depicted by the size of the circle. Sensitivity analyses showed directional concordance with the main MR approach for 91% and 75% of the associations calculated with weighted median MR and MR Egger, respectively. Missing MR parameters were due to an insufficient number of valid instruments (weighted median MR) or weak associations between instruments and exposure (MR Egger). Error bars and circles represent mean and 95% confidence intervals, respectively.
