# Supplementary material for: The genomic architecture of circulating cytokine levels points to drug targets for immune-related diseases
Source: Commun Biol. 2025 Jan 10;8:34. doi: 10.1038/s42003-025-07453-w (PMC11724035; doi:10.1038/s42003-025-07453-w)
Supplement: Supplementary file 3 — Description of Additional Supplementary Files [file 42003_2025_7453_MOESM3_ESM.pdf]

Description of Additional Supplementary Files

File name: Supplementary Data- Example code.R

Description: Example code for R studio statistical envirnment as referenced in the code availability section.

File name: Supplementary data S1

Description:

-Comparison of significant genomic loci across 3 proteomics assays

File name: Supplementary data S2

Description:

-significant trans- and cis-acting SNPs from GWAS meta-analysis

-significant trans-acting heterogenic SNPs at 0.1 from GWAS meta-analysis

-significant trans-acting heterogenic SNPs at 0.05 from GWAS meta-analysis

-genomic inflation factor Lambda, explained variance by genetic factors and Frequency of variants for individual cytokines

-Associations with cytokines from GWAS catalogue

File name: Supplementary data S3

Description:

-Fine-mapping results from Susie

File name: Supplementary data S4

Description:

-annotation results from MAGMA gene-based analysis

-tissue enrichment results from gene-property analysis

-pathway analysis results from MAGMA gene-set analysis

File name: Supplementary data S5

Description:

-associations between gene expression and circulating cytokine levels from inverse-variance weighted TWAS-MR analysis

-associations between gene expression and circulating cytokine levels from weighted median TWAS-MR analysis

-associations between gene expression and circulating cytokine levels from TWAS-MR Egger analysis

File name: Supplementary data S6

Description:

-LDSC cross-trait genetic correlations between the circulating levels

-associations between circulating cytokine concentrations from inverse-variance weighted MR analysis

-associations between circulating cytokine concentrations from weighted median MR analysis

-associations between circulating cytokine concentrations from MR Egger analysis

-associations between circulating cytokine concentrations excluding heterogenic variants (het P-value < 0.05) from inverse-variance weighted MR analysis

-associations between circulating cytokine concentrations excluding heterogenic variants (het P-value < 0.05) from weighted median MR analysis

-associations between circulating cytokine concentrations excluding heterogenic variants (het P-value < 0.05) from MR Egger analysis

File name: Supplementary data S7

Description:

-asscoations between cardiometabolic, allergic and autoimmune disease and cancer with circulating cytokine concentrations from inverse-variance weighted MR analysis

-asscoations between cardiometabolic, allergic and autoimmune disease and cancer with circulating cytokine concentrations from weighted median MR analysis

-asscoations between cardiometabolic, allergic and autoimmune disease and cancer with circulating cytokine concentrations from MR eggger analysis

-asscoations between cardiometabolic, allergic and autoimmune disease and cancer with circulating cytokine concentrations excluding heterogenic variants (Het P-value < 0.05) from inverse-variance weighted MR analysis

-asscoations between cardiometabolic, allergic and autoimmune disease and cancer with circulating cytokine concentrations excluding heterogenic variants (Het P-value < 0.05) from weighted median MR analysis

-asscoations between cardiometabolic, allergic and autoimmune disease and cancer with circulating cytokine concentrations excluding heterogenic variants (Het P-value < 0.05) from MR Egger analysis

File name: Supplementary data S8

Description:

-characteristics of the included GWAS databases

-characteristics of the included disease outcome

-ICD-9 and ICD-10 codes used to define outcomes in UKBB
