# Supplementary material for: The genomic architecture of circulating cytokine levels points to drug targets for immune-related diseases
Source: Commun Biol. 2025 Jan 10;8:34. doi: 10.1038/s42003-025-07453-w (PMC11724035; doi:10.1038/s42003-025-07453-w)
Supplement: Supplementary file 13 — Reporting summary [file 42003_2025_7453_MOESM13_ESM.pdf]

nature portfolio

Corresponding author(s): Marios Georgakis

Last updated by author(s): Nov 27, 2024

Reporting Summary

Nature Portfolio wishes to improve the reproducibility of the work that we publish. This form provides structure for consistency and transparency in reporting. For further information on Nature Portfolio policies, see our Editorial Policies and the Editorial Policy Checklist.

Statistics

For all statistical analyses, confirm that the following items are present in the figure legend, table legend, main text, or Methods section.

n/a	Confirmed	
☐	☒	The exact sample size (*n*) for each experimental group/condition, given as a discrete number and unit of measurement
☐	☒	A statement on whether measurements were taken from distinct samples or whether the same sample was measured repeatedly
☐	☒	The statistical test(s) used AND whether they are one- or two-sided<br>*Only common tests should be described solely by name; describe more complex techniques in the Methods section.*
☐	☒	A description of all covariates tested
☐	☒	A description of any assumptions or corrections, such as tests of normality and adjustment for multiple comparisons
☐	☒	A full description of the statistical parameters including central tendency (e.g. means) or other basic estimates (e.g. regression coefficient) AND variation (e.g. standard deviation) or associated estimates of uncertainty (e.g. confidence intervals)
☐	☒	For null hypothesis testing, the test statistic (e.g. *F*, *t*, *r*) with confidence intervals, effect sizes, degrees of freedom and *P* value noted<br>*Give P values as exact values whenever suitable.*
☒	☐	For Bayesian analysis, information on the choice of priors and Markov chain Monte Carlo settings
☒	☐	For hierarchical and complex designs, identification of the appropriate level for tests and full reporting of outcomes
☐	☒	Estimates of effect sizes (e.g. Cohen's *d*, Pearson's *r*), indicating how they were calculated

Our web collection on statistics for biologists contains articles on many of the points above.

Software and code

Policy information about availability of computer code

Data collection	no software was used to collect the data in the study
Data analysis	GTEx Portal: http://www.gtexportal.org/<br>NHGRI-EBI GWAS Catalog: http://www.ebi.ac.uk/gwas<br>FUMA Gene2Func: https://fuma.ctglab.nl/<br>RStudio 2023.12.1: https://www.rstudio.com/categories/rstudio-ide/<br>PLINK 1.90: https://www.cog-genomics.org/plink2<br>MAGMA 1.10: http://ctglab.nl/software/magma<br>METAL 2011-03-25: http://www.sph.umich.edu/csg/abecasis/Metal/<br>LDSC 1.0.1: https://github.com/bulik/ldsc.git

For manuscripts utilizing custom algorithms or software that are central to the research but not yet described in published literature, software must be made available to editors and reviewers. We strongly encourage code deposition in a community repository (e.g. GitHub). See the Nature Portfolio guidelines for submitting code & software for further information.

Data

Policy information about availability of data

All manuscripts must include a data availability statement. This statement should provide the following information, where applicable:

- Accession codes, unique identifiers, or web links for publicly available datasets
- A description of any restrictions on data availability
- For clinical datasets or third party data, please ensure that the statement adheres to our policy

Per-cytokine GWAS summary statistics are available at the EBI GWAS catalog (accession numbers GCST90428399 to GCST90428438).

Research involving human participants, their data, or biological material

Policy information about studies with human participants or human data. See also policy information about sex, gender (identity/presentation), and sexual orientation and race, ethnicity and racism.

Reporting on sex and gender	NA
Reporting on race, ethnicity, or other socially relevant groupings	NA
Population characteristics	NA
Recruitment	NA
Ethics oversight	NA

Note that full information on the approval of the study protocol must also be provided in the manuscript.

Field-specific reporting

Please select the one below that is the best fit for your research. If you are not sure, read the appropriate sections before making your selection.

☒ Life sciences ☐ Behavioural & social sciences ☐ Ecological, evolutionary & environmental sciences

For a reference copy of the document with all sections, see nature.com/documents/nr-reporting-summary-flat.pdf

Life sciences study design

All studies must disclose on these points even when the disclosure is negative.

Sample size	Our analyses are based on the eQTLGene Consortium data, Genotype Tissue Expression (GTEx) study, publicly available genome-wide association study (GWAS) results, UK10K reference panel and UKBiobank data. Sample sizes were hence determined by these studies. References to all GWAS analyzed in the paper are provided in Supplementary Table 8, and references to the GTEx, UK10K and UKBiobank resources are presented in our paper.
Data exclusions	We excluded 1 cytokine (IL-12) from the analyses, because different subunits of the protein were quantified in the two cohorts (IL-12p70 in YFS & FINRISK and IL-12p40 in deCODE).
Replication	Experimental replication was not attempted.
Randomization	NA
Blinding	NA

Reporting for specific materials, systems and methods

We require information from authors about some types of materials, experimental systems and methods used in many studies. Here, indicate whether each material, system or method listed is relevant to your study. If you are not sure if a list item applies to your research, read the appropriate section before selecting a response.

Materials & experimental systems

n/a	Involved in the study	
☒	☐	Antibodies
☒	☐	Eukaryotic cell lines
☒	☐	Palaeontology and archaeology
☒	☐	Animals and other organisms
☒	☐	Clinical data
☒	☐	Dual use research of concern
☒	☐	Plants

Methods

n/a	Involved in the study	
☒	☐	ChIP-seq
☒	☐	Flow cytometry
☒	☐	MRI-based neuroimaging

Plants

Seed stocks	NA
Novel plant genotypes	NA
Authentication	NA
